# Spread of TEM, VIM, SHV, and CTX-M *β*-Lactamases in Imipenem-Resistant Gram-Negative Bacilli Isolated from Egyptian Hospitals

**DOI:** 10.1155/2016/8382605

**Published:** 2016-03-31

**Authors:** El sayed Hamdy Mohammed, Ahmed Elsadek Fakhr, Hanan Mohammed El sayed, Said abd Elmohsen Al Johery, Wesam Abdel Ghani Hassanein

**Affiliations:** ^1^Department of Botany, Faculty of Science, Zagazig University, Zagazig 44511, Egypt; ^2^Department of Medical Microbiology & Immunology, Faculty of Medicine, Zagazig University, Zagazig 44511, Egypt

## Abstract

Carbapenem-resistant Gram-negative bacilli resulting from *β*-lactamases have been reported to be an important cause of nosocomial infections and are a critical therapeutic problem worldwide. This study aimed to describe the prevalence of imipenem-resistant Gram-negative bacilli isolates and detection of *bla*_VIM_, *bla*_TEM_, *bla*_SHV_, *bla*_CTX-M-1_, and *bla*_CTX-M-9_ genes in these clinical isolates in Egyptian hospitals. The isolates were collected from various clinical samples, identified by conventional methods and confirmed by API 20E. Antibiotic susceptibility testing was determined by Kirby-Bauer technique and interpreted according to CLSI. Production of *bla*_VIM_, *bla*_TEM_, *bla*_SHV_, and *bla*_CTX-M_ genes was done by polymerase chain reaction (PCR). Direct sequencing from PCR products was subsequently carried out to identify and confirm these *β*-lactamases genes. Out of 65 isolates, (46.1%) Escherichia coli, (26.2%) Klebsiella pneumoniae, and (10.7%) Pseudomonas aeruginosa were identified as the commonest Gram-negative bacilli. 33(50.8%) were imipenem-resistant isolates. 22 isolates (66.7%) carried *bla*_VIM_, 24(72.7%) had *bla*_TEM_, and 5(15%) showed *bla*_SHV_, while 12(36%), 6(18.2%), and 0(0.00%) harbored *bla*_CTX-M-1_, *bla*_CTX-M-9_, and *bla*_CTX-M-8/25_, respectively. There is a high occurrence of *β*-lactamase genes in clinical isolates and sequence analysis of amplified genes showed differences between multiple SNPs (single nucleotide polymorphism) sites in the same gene among local isolates in relation to published sequences.

## 1. Introduction

Gram-negative bacilli are a heterogeneous group of Gram-negative bacteria that are common commensals, infectious agents and also sometimes referred to as “nightmare bacteria” [1]. Hospital acquired infections due to Gram-negative bacilli are a leading cause of morbidity and mortality worldwide [2].

Carbapenem, a member of the *β*-lactam family, has a broad spectrum of activity and is stable to most *β*-lactamases. These properties make carbapenem an important therapeutic option for treating serious infections involving resistant strains of Enterobacteriaceae, anaerobes,* Pseudomonas aeruginosa*, and* Acinetobacter *spp. [3], although carbapenems, including imipenem and meropenem, are often used as “antibiotics of last resort” when patients with infections become severely ill or are suspected of harboring resistant bacteria [4]. However, carbapenem-resistant Gram-negative bacilli isolates were increasingly reported worldwide [5].

This resistance may be attributed to presence of metallo-*β*-lactamase in bacteria such as IMP (Imipenemase), VIM (Verona-Integron metallo-*β*-lactamase) [6], and extended spectrum *β*-lactamases (ESBLs) such as SHV, TEM, and CTX-M [7].

For establishment of appropriate antimicrobial therapy and control of the spread of drug resistant Gram-negative bacilli, the PCR-based detection methods of resistant genes show the bioinformatics analysis of their molecular diversity and evolution becoming increasingly important [8].

This work aimed to study distribution of imipenem-resistant Gram-negative isolates and shed focused light on some genes encoding beta-lactamase enzymes responsible for such resistance in Zagazig University Hospitals in Egypt.

## 2. Material and Methods

### 2.1. Bacterial Isolates

Clinical isolates of Gram-negative bacilli including* Escherichia coli* (*n* = 30),* Klebsiella pneumoniae* (*n* = 17),* Pseudomonas aeruginosa* (*n* = 7),* Proteus mirabilis* (*n* = 2),* Citrobacter freundii* (*n* = 1),* Acinetobacter baumanii* (*n* = 3), and* Enterobacter cloacae* (*n* = 4) were collected from blood, urine, pus, and sputum specimens from hospitalized patients in Zagazig University Hospitals in Egypt from January 2013 to March 2014. These clinical samples were processed by plating on blood agar and MacConkey agar [9]. A growth temperature of 44°C was used sometimes to confirm the identity of these isolates and the identified strains were stored in glycerol (20% V/V) at 70°C and subcultured several times to be viable. All isolates were identified by standard biochemical tests [10] and confirmed by API 20E (BioMérieux, Marcy l'Étoile, France).

### 2.2. Antibiotic Susceptibility Testing

The susceptibility testing of studied isolates was performed by disc diffusion method (modified Kirby-Bauer method) using Muller-Hinton agar (Becton Dickinson, MA, USA) and interpreted according to the Clinical Laboratory Standard Institute (CLSI) guidelines [11]. The antibiotic disks used imipenem (IPM, 10 *μ*g), amikacin (AK, 30 *μ*g), ciprofloxacin (CIP, 5 *μ*g), piperacillin (PRL, 100 *μ*g), cefoperazone/sulbactam (CES, 10 + 5 *μ*g), cefoxitin (FOX, 30 *μ*g), and cefotaxime (CTX, 30 *μ*g) which were placed 15 mm away from the central disc and the plates were incubated for about 18–24 hrs at 37°C.

### 2.3. Molecular Detection of *β*-Lactamase Genes by PCR

For detection of *β*-lactamase genes responsible for imipenem-resistance, rapid genomic DNA was prepared from about five colonies heated in 100 mL distilled water (95°C for 10 min) followed by a centrifugation step of cell suspension at 12.000 rpm for 5 min; then supernatant was taken as a source of template DNA. PCR amplification was carried out by using DNA thermal cycler (Biometra, Singapore) using a specific primer for *bla*_VIM_, *bla*_TEM_, *bla*_SHV_, *bla*_CTX-M-1_, *bla*_CTX-M-9_, and *bla*_CTX-M-8/25_ (Table 1), in a 50 *μ*L volume containing 10x PCR buffer, 2 mM deoxynucleoside triphosphates, 3.4 pmol of each primer, 2.5 mM MgCl_2_, 1 U Taq DNA polymerase, and 1 *μ*L of genomic DNA [12]. Amplification was carried out as follows: initial denaturation at 94°C for 10 minutes, followed by 40 cycles of DNA denaturation at 94°C for 40 seconds, primer annealing at 60°C for 40 seconds and primer extension at 72°C for 1 minute, and a final elongation step at 72°C for 7 minutes. The annealing temperature was optimal at 55°C instead of 60°C for amplification of *bla*_VIM_. Amplicons were then visualized after running in 2% agarose gel at 100 V for 30 mins. A 50–1000 bp DNA ladder (USA) was used as a size marker. Finally, PCR products were purified with innuPREP PCRpure kit (Analytik Jena, Germany) and subjected to direct sequencing via GATC Company by use of ABI 3730xl DNA sequencer.

### 2.4. Bioinformatics and Sequences Analysis

The obtained chromatogram sequencing files were inspected and corrected using the software application Chromas 2.3 (Technelysium, Helensvale, Australia) and JalView (2.8).

The sequences obtained from our samples were aligned with GenBank sequences. The phylogenetic tree for each sequence was obtained by performing neighbor-joining analysis of the alignment of sequences with reference strains (accession numbers/country of origin) that were retrieved from GenBank. The studied strains were marked by the sign [■]. Meanwhile, the reference sequences were marked by the sign [▲].

The BLAST and FASTA programs of the National Center for Biotechnology Information (http://blast.ncbi.nlm.nih.gov/Blast.cgi) were used to search databases for similar nucleotide sequences [13]. Multiple sequence alignments of the nucleic acid were carried out using the ClustalW program. The statistical analysis was performed using SPSS version 20.0, *χ* ^2^ = chi-square test, and *P* values < 0.05 were considered significant.

## 3. Results

### 3.1. Isolation and Identification

The present study was conducted on 65 screened isolates of Gram-negative bacilli, obtained from 108 various clinical samples such as urine, blood, pus, and sputum, where 41 (78.8%) were isolated from urine, 12 (66.6%) from blood, 14 (48.2%) from respiratory secretions, and 2 (22.2%) from pus.* Escherichia coli* (46.1%) was the most commonly isolated organism among Gram-negative bacilli, followed by* Klebsiella pneumoniae* (26.2%),* Pseudomonas aeruginosa* (10.7%),* Enterobacter cloacae* (6.1%),* Proteus mirabilis* (3.07%), and* Acinetobacter baumanii* (4.6%) while* Citrobacter freundii* and* Proteus vulgaris* gave 1.5%. The isolation of Gram-negative bacilli isolates was significantly higher in patients with trauma (*P* < 0.001), those hospitalized for more than 7 days (*P* < 0.001), and those with ICU admission (*P* < 0.001) harboring risk factors for acquiring Gram-negative bacilli infection.

### 3.2. The Antibiotic Susceptibility Testing

**Figure 1:**
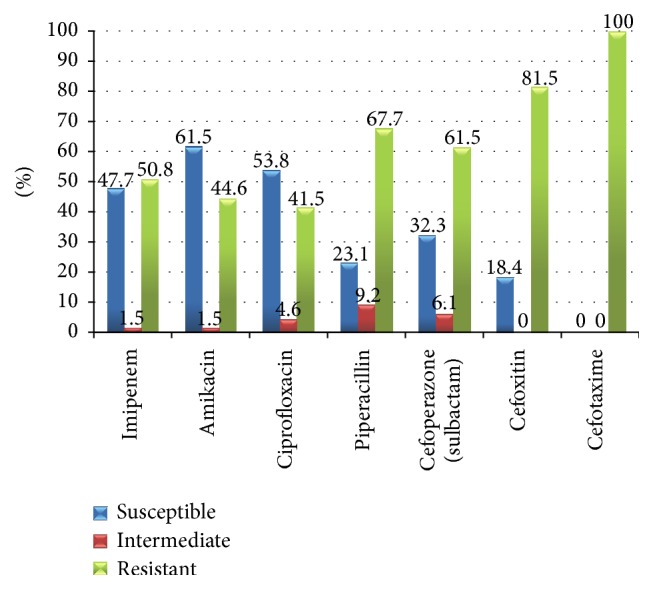
Antimicrobial susceptibility patterns of all 65 Gram-negative bacilli isolates.

It was shown in Figure 1, as observed, that 40 (61.5%) were susceptible to amikacin, 35 (53.8%) were susceptible to ciprofloxacin, 31 (47.7%) were susceptible to imipenem, and 21 (32.3%) were susceptible to cefoperazone/sulbactam. However, all isolates were resistant to cefotaxime (100%), 53 (81.5%) of isolates were resistant to cefoxitin, 44 (67.7%) were resistant to piperacillin, 40 (61.5%) were resistant to cefoperazone/sulbactam, 33 (50.8%) were resistant to imipenem followed by 24 (44.6%) resistant to amikacin, and 27 (41.5%) were resistant to ciprofloxacin.

### 3.3. PCR Assay Results

**Figure 2:**
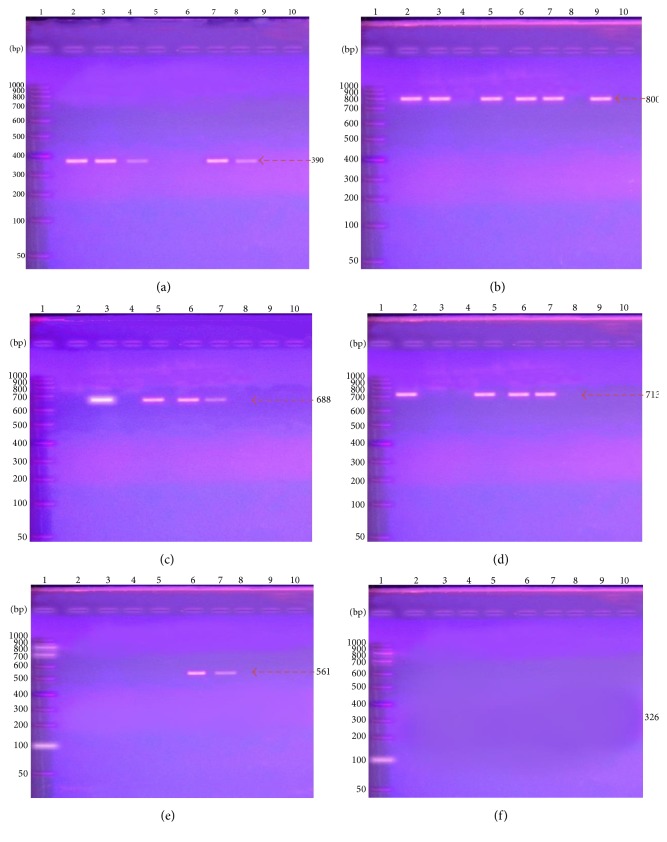
Presence and absence of *β*-lactamase genes by PCR amplification in some isolated samples. (a) The existence of VIM amplification fragment (390 bp). (b) The amplification of TEM fragments (800 bp). (c) The amplification of CTX-M-1 (688 bp). (d) The presence of SHV (713 bp) in some samples. (e) The presence of CTX-M-9 (561 bp). (f) No amplification was shown with CTX-M-8/25 primer at 326 bp.

As shown in Table 6, of 33 imipenem-resistant Gram-negative bacillistrains, the results of PCR amplification products of *β*-lactamases genes showed that 66.7% of isolates carried *bla*_VIM_ at 390 bp (Figure 2(a)), 72.7% had *bla*_TEM_ at 800 bp (Figure 2(b)), 36.0% harbored *bla*_CTX-M-1_ at 688 bp (Figure 2(c)), and 15.0% showed *bla*_SHV_ (Figure 2(d)), while 18.2% (Figure 2(e)), 0.00% (Figure 2(f)) harbored *bla*_CTX-M-9_, *bla*_CTX-M-8/25_, respectively. Sequencing confirmed presence of these *β*-lactamase genes; the GenBank nucleotide sequence accession numbers for the sequences studied are detailed. Aligning of the obtained sequences with those of reference strains in GenBank confirmed the correct identification of *bla*_VIM_, *bla*_TEM_, *bla*_SHV_, and *bla*_CTX-M_ genes by PCR.

### 3.4. Sequences Analysis and Polymorphism

#### 3.4.1. The Analysis of VIM Gene (*bla*_VIM1,2_)

The sequence of the purified product of VIM gene (*bla*_VIM1,2_) was compared with homologous GenBank sequences using BLAST program and resulted in significant similarity to many metallo-*β*-lactamases genes of different bacterial strains.

**Figure 3:**
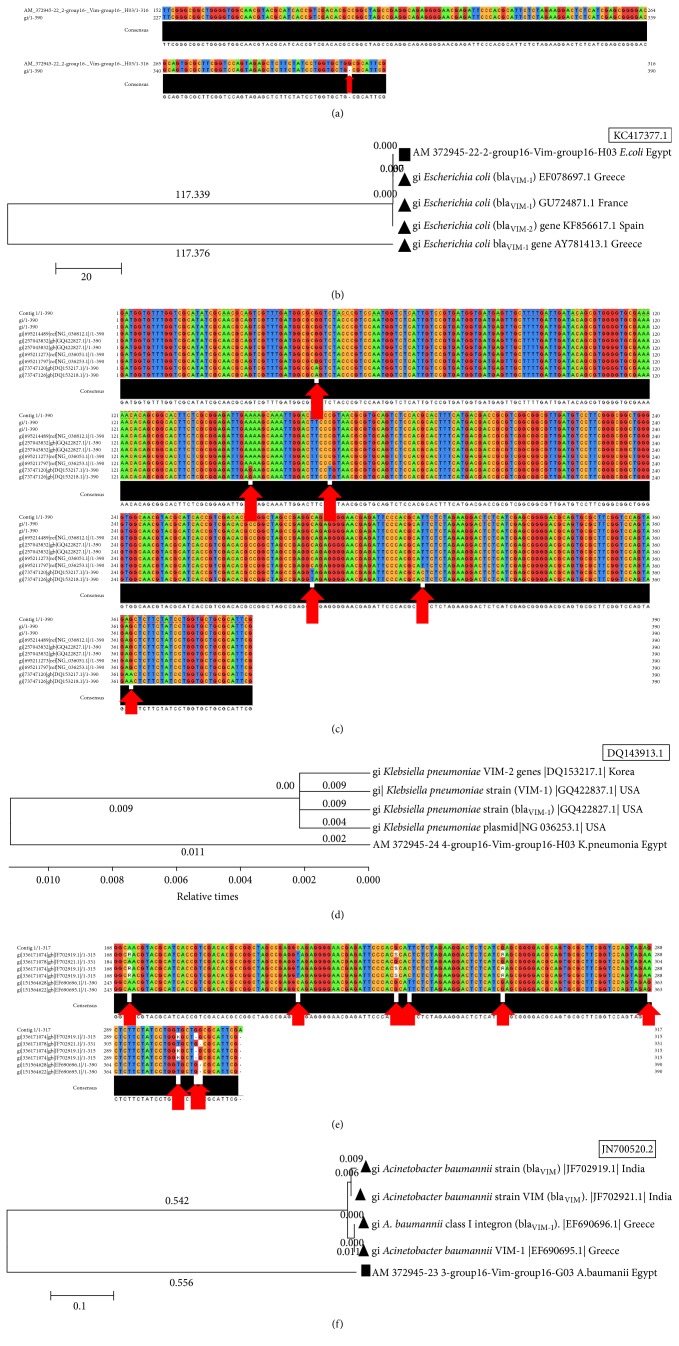
The multiple sequences alignments and trees of VIM gene in different species. (a) VIM gene in* E. coli *strains isolated from Zagazig University (ZU) Hospitals in comparison with published VIM gene of* E. coli *strains from GenBank. (b) A tree of VIM sequence of* E. coli *isolated from ZU Hospitals and published homologs in GenBank. (c) The multiple sequences alignments of VIM gene in* K. pneumoniae *strains isolated from ZU Hospitals in comparison with published VIM gene in* K. pneumoniae *strains from GenBank. (d) Phylogenetic tree of VIM sequence of* K. pneumoniae *isolated from ZU Hospitals and published sequences in GenBank. (e) The multiple sequences alignments of VIM gene in* A. baumanii *strains isolated from ZU Hospitals in comparison with published VIM gene in* A. baumanii *strains from GenBank. (f) Phylogenetic tree of VIM sequence of* A. baumanii *isolated from ZU Hospitals and published sequences in GenBank.

*(1) VIM Gene (bla*_*VIM1,2*_ *) in Escherichia coli Strains*. The pairwise sequences alignments of resulting VIM gene (*bla*_VIM1,2_) in* E. coli* strains, isolated from Zagazig University (ZU) Hospitals, in comparison with published VIM gene in* E. coli* strains from GenBank, for example (*E. coli *KC417377.1), showed single common SNP (single nucleotide polymorphism) sites between the different strains. The SNPs position was indicated in position 382 in the Egyptian strains (Figure 3(a)). The phylogenetic tree of VIM gene sequence in* E. coli* strains, isolated from Zagazig University Hospitals, and published homologous sequences in GenBank showed different degrees of dis/similarity between the different strains (Figure 3(b)). It was interesting to detect that both strains, the most similar and most dissimilar strains, were from the same country, Greece, indicating biodiversity in the same geographical location.

*(2) VIM Gene (bla*_*VIM1,2*_ *) in Klebsiella pneumoniae Strains*. The VIM gene isolated from* K. pneumoniae* strains in Zagazig University Hospitals was compared with published VIM gene in* K. pneumoniae* strains from GenBank (e.g.* K. pneumoniae* DQ143913.1). The results showed 6 different common SNPs between the different strains. The SNPs positions were indicated in 45, 150, 168, 284, 309, and 363 (Figure 3(c)). The phylogenetic tree of VIM gene sequence in* K. pneumoniae* strains, isolated from ZU Hospitals, and published homologous sequences in GenBank showed different degrees of dis/similarity between the different strains with many unique sequences in the Egyptian strain (Figure 3(d)).

*(3) VIM Gene (bla*_*VIM1,2*_ *) in Acinetobacter baumanii Strains*. The sequence of purified product of VIM gene (*bla*_VIM1,2_) from* Acinetobacter baumanii* strain was compared with the GenBank sequence using BLAST program. Interestingly, it was revealed that there was a single strain present in GenBank which is completely different from the studied strains and this is not matching with our study (e.g.,* Staphylococcus phage* StB12, complete genome). The sequence alignment showed 9 different common SNPs between the different strains. These positions were indicated in 246, 284, 306, 309, 330, 363, 378, 382, and 383 (Figure 3(e)). The phylogenetic tree of VIM sequence of* A. baumanii* isolated from ZU Hospitals and published ones in GenBank showed dissimilarity between Egyptian strains and others (Figure 3(f)).

#### 3.4.2. Sequence Analysis of TEM Gene (*bla*_VIM1,2_)

Sequences of the purified product of VIM gene (*bla*_VIM1,2_) were compared with homologous counterpart GenBank database using BLAST program and resulted in significant similarity to many metallo-*β*-lactamases genes of different bacterial strains.

**Figure 4:**
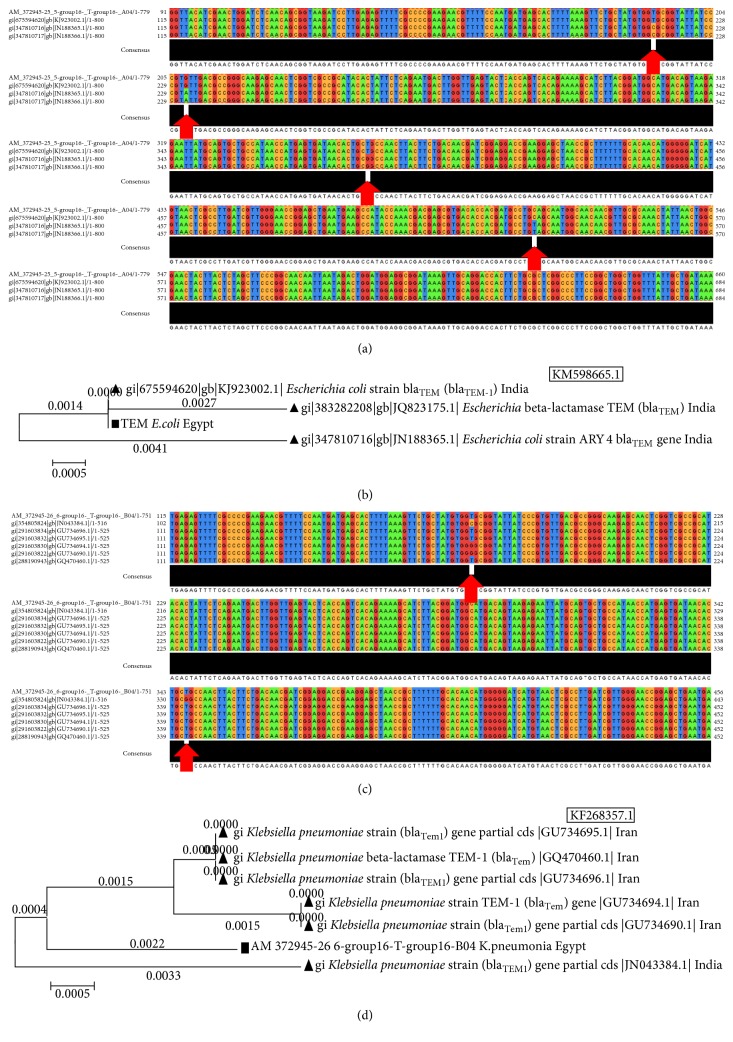
The multiple sequences alignments and trees of TEM gene (*bla*_TEM1,2_) in different bacterial species. (a) TEM gene in* E. coli *strains isolated from ZU Hospitals in comparison with published TEM gene of* E. coli *strains from GenBank. (b) A phylogenetic tree of TEM gene of* E. coli *isolated from ZU Hospitals and published homologous ones in GenBank. (c) The multiple sequences alignments of gene in* K. pneumoniae *strains isolated from ZU Hospitals in comparison with published TEM gene in* K. pneumoniae *strains from GenBank. (d) Phylogenetic tree of sequence of* K. pneumoniae* isolated from ZU Hospitals and published homologous ones in GenBank.

*(1) TEM Gene (bla*_*TEM1,2*_ *) of Escherichia coli*. The TEM gene sequences of* E. coli* strains, isolated from ZU Hospitals, were aligned with sequences of published TEM genes in* E. coli* strains from GenBank (e.g.,* E. coli* KM598665.1). The resulting alignments showed 4 different common SNP sites between the different strains. The SNPs positions were indicated commonly in 216, 232, 385, and 433 (Figure 4(a)). Phylogenetic tree was constructed from TEM gene (*bla*_TEM1,2_) sequence of* Escherichia coli* strains isolated from ZU Hospitals and published homologous sequences in GenBank (Figure 4(b)) showing similarity degree between Egyptian and Indian strains.

*(2) TEM Gene (bla*_*TEM1,2*_ *) of Klebsiella pneumonia*. Multiple sequences alignments of TEM gene in* K. pneumoniae* strains, isolated from ZU Hospitals, were compared with other published TEM genes in* K. pneumoniae* strains from GenBank (e.g.,* K. pneumoniae* KF268357.1). (Figure 4(c)) showed 2 different common SNPs between different strains. The SNPs positions were indicated in 174 and 343. A phylogenetic tree of TEM sequence of* K*.* pneumoniae* isolated from Zagazig University Hospitals and other published ones in GenBank showed the degree of similarity between strains where Egyptian strain was dissimilar to that of Iranian and Indian strains (Figure 4(d)).

#### 3.4.3. The Analysis of SHV Gene (*bla*_SHV1_) in* Klebsiella pneumoniae* Strains

**Figure 5:**
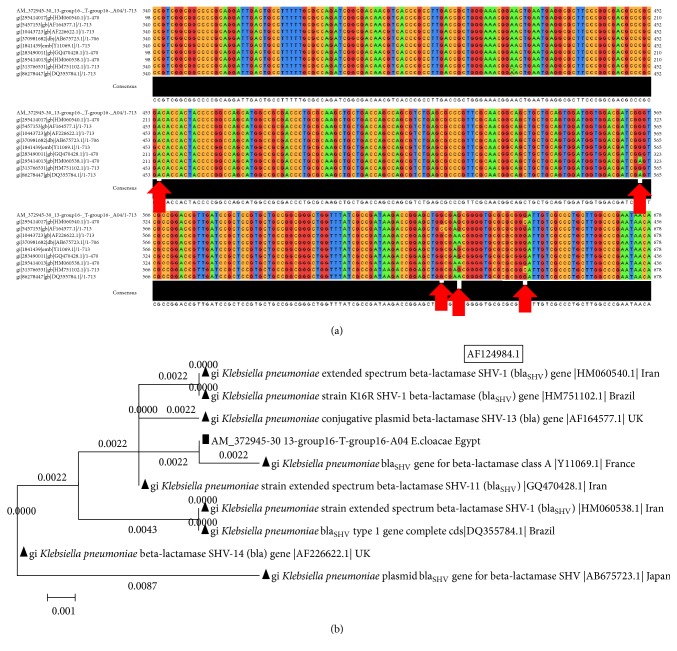
The multiple sequences alignments and trees of SHV gene in different species. (a) SHV gene in* K. pneumoniae *strains isolated from ZU Hospitals in comparison with published SHV gene of* K. pneumoniae *strains from GenBank. (b) Phylogenetic tree of SHV sequence of* K. pneumoniae *isolated from ZU Hospitals and published homologous ones in GenBank.

The pairwise sequences alignments of resulting SHV gene (*bla*_SHV1_) in* K. pneumoniae* strains, isolated from Zagazig University Hospitals, in comparison with published SHV gene in* K. pneumoniae* strains from GenBank using BLAST program (e,g.,* K. pneumoniae* AF124984.1) showed five common SNPs between the different strains. The SNPs position was indicated in 454, 563, 631, 635, and 650 in Egyptian strains (Figure 5(a)). The phylogenetic tree of SHV gene in this case showed that Egyptian strain was more similar to France strain (Figure 5(b)).

#### 3.4.4. The Analysis of CTX-M-1 Gene (*bla*_CTX-M-1_)

Sequences of the purified product of CTX-M-1 gene (*bla*_CTX-M-1_) were compared with homologous counterpart GenBank database using BLAST program and resulted in significant similarity to many metallo-*β*-lactamases genes of different bacterial strains.

**Figure 6:**
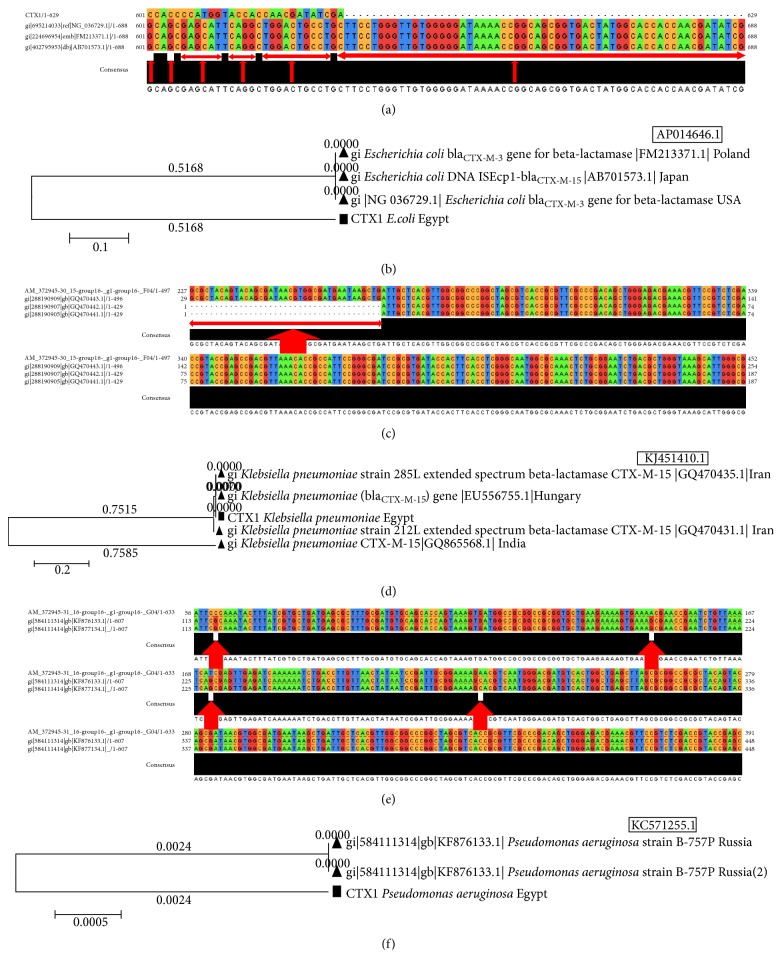
The multiple sequences alignments and trees of CTX-M-1 gene in different species. (a) CTX-M-1 gene in* E. coli *strains isolated from ZU Hospitals in comparison with published CTX-M-1 gene of* E. coli *strains from GenBank. (b) A tree of CTX-M-1 sequence of* E. coli *isolated from ZU Hospitals and published homologs in GenBank. (c) The multiple sequences alignments of CTX-M-1 gene in* K. pneumoniae *strains isolated from ZU Hospitals in comparison with published CTX-M-1 gene in* K. pneumoniae *strains from GenBank. (d) Phylogenetic tree of CTX-M-1 sequence of* K. pneumoniae *isolated from ZU Hospitals and published homologous sequences in GenBank. (e) Multiple sequences alignments of CTX-M-1 gene in* P. aeruginosa *strains isolated from ZU Hospitals in comparison with published CTX-M-1 gene in* P. aeruginosa *strains from GenBank. (f) Phylogenetic tree of CTX-M-1 sequence of* P. aeruginosa *isolated from ZU Hospitals and published homologs in GenBank.

*(1) CTX-M-1 Gene (bla*_*CTX-M-1*_ *) of Escherichia coli Strains*. The sequence of the purified product of CTX-M-1 gene from* Escherichia coli* strains was compared with the GenBank sequence using BLAST program. Interestingly, it was revealed that there were strains present in GenBank which are completely different from the studied strains and this is not matching with our study (e.g.,* Pseudomonas aeruginosa* DNA, AP014646.1). The sequence alignment showed 81 different common SNPs between the different strains. The SNPs positions were indicated in 601, 604 → 615, 617 → 626, and 628 → 688 (Figure 6(a)). The phylogenetic tree of CTX-M-1 sequence of* E. coli* isolated from Zagazig University Hospitals and published homologous ones in GenBank showed dissimilarity degree between Egyptian and other universal strains (Figure 6(b)).

*(2) CTX-M-1 Gene (bla*_*CTX-M-1*_ *) of Klebsiella pneumoniae Strains*. The alignment in this case showed 39 different common SNPs between the different strains. The SNPs positions were indicated in positions 1 → 39 (Figure 6(c)). A phylogenetic tree of CTX-M-1 gene sequence in* K. pneumoniae* strains, isolated from ZU Hospitals, and published homologous sequences in GenBank showed different degrees of dis/similarity between the different strains with many unique sequences in Egyptian strain (Figure 6(d)).

*(3) CTX-M-1 Gene (bla*_*CTX-M-1*_ *) of Pseudomonas aeruginosa Strains*. The CTX-M-1 gene isolated from* P. aeruginosa *strains in Zagazig University Hospitals was compared using BLAST program with published CTX-M-1 gene in* P. aeruginosa *strains from GenBank (e.g.,* P.aeruginosa* KC571255.1). The results showed 4 different common SNPs between the different strains. The SNPs positions were indicated in 117, 206, 228, and 283 (Figure 6(e)). A phylogenetic tree showed similarity degree between Egyptian and Russian strains (Figure 6(f)).

#### 3.4.5. The Analysis of CTX-M-9 Gene (*bla*_CTX-M-9_) in* Escherichia coli* Strains

**Figure 7:**
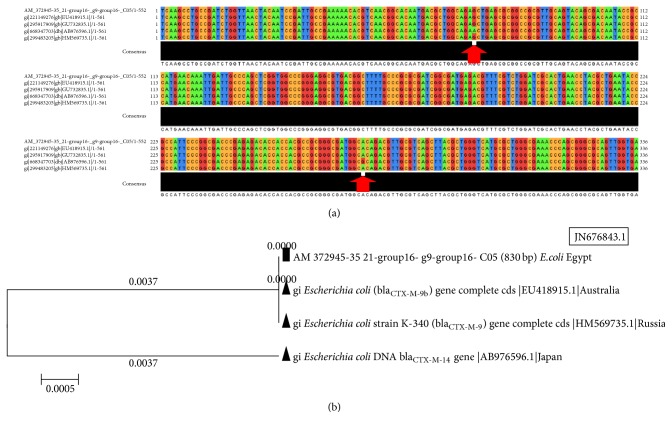
The multiple sequences alignments and tree of CTX-M-9 gene in different species. (a) CTX-M-9 gene in* E. coli *strains isolated from ZU Hospitals in comparison with published CTX-M-9 gene of* E. coli *strains from GenBank. (b) Phylogenetic tree of CTX-M-9 sequence of* E. coli *isolated from ZU Hospitals and published homologous ones in GenBank.

The CTX-M-9 gene isolated from* E. coli* strains in Zagazig University Hospitals was compared with published CTX-M-1 gene in* P. aeruginosa* strains from GenBank using BLAST program (e.g.,* E. coli* JN676843.1). The multiple sequences alignments showed 2 different common SNPs in positions 74 and 272 (Figure 7(a)). The phylogenetic tree of CTX-M-9 gene sequence in* E. coli* strains, isolated from ZU Hospitals, and published homologous sequences in GenBank showed different degrees of dis/similarity between the different strains and many unique sequences in the Egyptian strain similar to that of Russia and Australia and dissimilar to that of Japan (Figure 7(b)).

## 4. Discussion

The Gram-negative bacilli are among the most important causes of serious nosocomial and community-onset bacterial infections in humans and antimicrobial resistance has become a global threat to effective health care delivery [14]. However, carbapenem-resistant Gram-negative bacilli have been increasingly reported worldwide [4]. Various acquired carbapenemases have been identified in the last years, belonging to either acquired metallo-beta-lactamases (IMP, VIM, SPM, GIM, NDM, and DIM types) or class A (KPC and GES) and class D *β*-lactamase OXA-48 [15].

In the present study, prevalence of *β*-lactamases-producing isolates was found in Table 2. Different studies carried out by other workers in various parts of the world show quite variable results. In a study carried out, the frequency of beta-lactamases-producing isolates was urine (61%), followed by blood cultures (38%), wound swabs (13%), and tracheal aspirates (5%) (*P* < 0.001) [16]. And this is similar to our study. By contrast, Shanthi and Sekar [17] in India reported that Gram-negative isolates were obtained from the respiratory tract (41.8%) followed by urinary tract (25.5%), wound (20%), and blood (12.7%). Also, pus was the most common specimen accounting for 21% followed by tracheal aspirate (17%), sputum (16%), urine (11%), and blood (7%) [18].

Various risk factors of *β*-lactamases have been implicated in selection and spread producing strains from various clinical samples.

In accordance with distribution of Gram-negative bacilli, (46.1%)* Escherichia coli* and (26.2%)* Klebsiella pneumoniae* isolates followed by (10.7%)* P*.* aeruginosa* were identified as the commonest isolates among Gram-negative bacilli (Table 3) and this coincided with that concluded by Sahu et al. [19] who found that 58% were identified as* Escherichia coli* followed by 27.7%* Klebsiella pneumoniae* and 15%* Pseudomonas aeruginosa *in Udaipur, Rajasthan. In a study by Vipin et al. [20] 52 (58.42%) isolates of* Escherichia coli *were found to be the most common organisms in Allahabad followed by* Klebsiella pneumoniae *(20.22%),* Pseudomonas aeruginosa *(12.35%),* Proteus vulgaris *(3.37%),* Proteus mirabilis* (2.24%), and* Enterobacter cloacae *(2.24%). Also, in India, Sankarankutty and Kaup [21] documented the same result in that 58.42% isolates of* Escherichia coli *were found to be the most common organisms followed by* Klebsiella pneumoniae *(20.22%),* Pseudomonas aeruginosa *(12.35%),* Proteus vulgaris *(3.37%),* Proteus mirabilis *(2.24%), and* Enterobacter aerogenes *(2.24%).

But our study disagreed with that published by Aboderin et al. [22] who reported that* Pseudomonas aeruginosa *recorded the highest prevalence followed by* Klebsiella pneumoniae* and ESBL producers, whereas frequency among* E. coli* isolates was much lower than* Klebsiella pneumoniae*. Hence, prevalence of pathogens often varies dramatically between communities according to geography, hospitals in the same community and among different patient populations in the same hospital.

In this study, risk factors associated with isolation of Gram-negative bacilli isolates were shown in Table 4. And this was matched with that reported by Kumar et al. [23] who exhibited major risk factors such as prolonged hospitalization > 8 days, previous antibiotic use, trauma, and mechanical ventilation which may contribute to the mortality.

In Turkey, Aktas et al. [24] reported risk factors for acquisition including prolonged hospitalization, an ICU stay, ventilator usage, previous use of carbapenem antibiotics, and the presence of underlying diseases and this is compatible with our research. But in Brazil, Tuon et al. [25] documented that there was statistical significance in isolation of* Klebsiella pneumoniae* isolates according to age (*P* = 0.005) and mechanical ventilation (*P* = 0.003), while trauma (*P* = 0.87) and ICU stay (*P* = 0.25) had a statistical significance as major risk factors.

The importance of these risk factors lies in the epidemiological implications at the hospital level because the results suggest a probable nosocomial transmission of the infection.

Resistance pattern among nosocomial bacterial pathogens may vary widely from country to country at any time and within the same country over time [26].

In our study, all the isolates were resistant to cefotaxime (100%) and displayed unusually high level of imipenem-resistance (50.8%) isolates with MICs ranging from 15.6 to 250 *μ*g/mL (Table 5) (*P* < 0.001). In Egypt, this was parallel to that reported by Mohamed and Raafat [27] who reported (52.2%) imipenem-resistance among isolates, (100%) cefotaxime resistance, (55%) susceptible-ciprofloxacin, and (70%) susceptibility to amikacin. In the Middle East, the occurrence of imipenem-resistant Gram-negative bacilli is alarmingly elevated. Another similar study showed that all 84* Klebsiella pneumoniae* isolates exhibited resistance to imipenem with MICs ranging from 4 to >32 *μ*g/mL in a Greek hospital [28].

Another dissimilar study was shown in Saudi Arabia, where the susceptibility rate of Gram-negative organisms isolated from a tertiary care hospital to imipenem was reported to be as low as 10% [29]. Galani et al. [30] reported that* Klebsiella pneumoniae *and* Escherichia coli *isolates were found to be susceptible to imipenem in routine susceptibility disk diffusion tests. Other authors observed that all beta-lactamases producers of* Escherichia coli*,* Klebsiella pneumoniae,* and* Pseudomonas aeruginosa* were susceptible to imipenem showing coresistance to other antibiotics of aminoglycosides, fluoroquinolones, and others [17]. The extensive use of carbapenems in some locations has likely created a selective antibiotic pressure which in turn has resulted in an increased prevalence of carbapenem-resistant Gram-negative isolates.

The carbapenem resistance due to production of *β*-lactamases has a potential for rapid dissemination, since it is often plasmid-mediated [2]. Consequently, rapid detection of *β*-lactamases is necessary to initiate effective infection control measures to prevent their uncontrolled spread in clinical settings. In Egyptian hospitals the *β*-lactamases presence was confirmed by PCR amplification.

In our study, the percentage of *bla*_VIM_, *bla*_TEM_, *bla*_SHV_, and *bla*_CTX-M_ genes among Gram-negative bacilli isolates was shown in Table 6. As regards *bla*_VIM_ gene detected in our results it is similar to that *bla*_VIM_ gene encoding MBL among the isolates of* P. aeruginosa* (61.3%) in Tehran hospitals [31]. On the contrary, *bla*_VIM_ genes were not detected among the studied Gram-negative isolates in other Tehran hospitals [32]. TEM, SHV, and CTX-M genes are the most common plasmid-mediated lactamases often found in Enterobacteriaceae and* P. aeruginosa* [33]. In Iran, Eftekhar et al. [34] showed 69.3%  *bla*_TEM_ and 31.37%  *bla*_CTX-M-1_ genes among Gram-negative isolates and this agreed with our study. Also, presence of *bla*_CTX-M1,9_, *a*_SHV_, and *bla*_TEM_ genes among tested rods of the Enterobacteriaceae family was revealed by Ojdana et al. [35]. Another study reported by Cuzon et al. [36] harbored similar results regarding carbapenem-resistant Gram-negative isolates carrying TEM, SHV, CTX-M-1, and CTX-M-9 genes.

In Switzerland, Zurfluh et al. [37] mentioned that the isolates of Enterobacteriaceae were further screened for* bla* genes encoding SHV, TEM, CTX-M group 1, CTX-M group 2, or CTX-M group 9 enzymes with (23%), (71.3%), (39.2%), (20.1%), and (15.6%), respectively.

Other authors noted different observations in that clinical isolates of* Escherichia coli* and* Pseudomonas aeruginosa* carried *bla*_CTX-M-types_ which was the most common (95.8%) followed by *bla*_TEM_ (29.2%), *bla*_SHV_ (7.3%), and *bla*_VIM_ (12.5%) in Nepal [38]. These observations contribute to the knowledge of the epidemiology of VIM, TEM, SHV, and CTX-M-producing Gram-negative isolates that have now become endemic in major hospitals in Egypt. Continuous monitoring, proper infection control, and surveillance and prevention practices will limit the further spread of these infections within these hospitals and clinical settings.

Multiple sequences analysis is used in such biological studies to extract important phylogenetic and evolutionary information using different scoring matrices (BLOSUM62 for BLAST, BLOSUM50 for SEARCH and FASTA) [39].

## 5. Conclusion

There is a high prevalence of *β*-lactamase genes in our clinical isolates that are responsible for such resistance. Hence, it is essential to report *β*-lactamases production along with routine sensitivity reports, which will help the clinician in prescribing proper antibiotics. Also, the sequence analysis of amplified genes showed differences between multiple SNPs in the same gene among different local isolates and with internationally published sequences. In the end, it has been felt that there is a need to formulate strategies to detect and prevent the emergence of *β*-lactamases producing strains for the effective treatment of infections which are caused by them.

## Figures and Tables

**Table 1:** Primer name, primer sequences, and expected amplicon size of amplified DNA products.

PCR name	*β*-lactamase targeted	Primer name	Sequence (5′-3′)	Amplicon size (bp)
VIM	VIM variants including VIM-1 and VIM-2	VIM-for	GATGGTGTTTGGTCGCATA	390
		VIM-rev	CGAATGCGCAGCACCAG	

TEM	TEM variants including TEM-1 and TEM-2	TSO-T-for	CATTTCCGTGTCGCCCTTATTC	800
		TSO-T-rev	CGTTCATCCATAGTTGCCTGAC	

SHV	SHV variants including SHV-1	TSO-S-for	AGCCGCTTGAGCAAATTAAAC	713
		TSO-S-rev	ATCCCGCAGATAAATCACCAC	

CTX-M group 1	Variants of CTX-M group 1 including CTX-M-1, CTX-M-3, and CTX-M-15	CTXMGp 1-for	TTAGGAARTGTGCCGCTGYA^b^	688
		CTXMGp 1.2 rev	CGATATCGTTGGTGGTRCCAT^b^	

CTX-M group 9	Variants of CTX-M-9 including CTX-M-9 and CTX-M-14	CTX-9-F	TCAAGCCTGCCGATCTGGT	561
		CTX-9-R	TGATTCTCGCCGCTGAAG	

CTX-M group 8/25	CTX-M-8, CTX-M-25, CTX-M-26, and CTX-M-39 to CTX-M-41	CTX-8/25-F	AACRCRCAGACGCTCTAC^b^	326
		CTX-8/25-R	TCGAGCCGGAASGTGTYAT^b^	

^b^Y = T or C; R = A or G; S = G or C.

**Table 2:** The distribution of Gram-negative bacilli isolates in each clinical specimen.

Clinical specimens		Gram-negative bacilli isolates	
Type	Number	Number	(%)
Urine	52	41	78.8
Blood	18	12	66.6
Sputum	29	10	34.5
Pus	9	2	22.2

Total	**108**	**65**	**(60.2%)**

*χ* ^2^ = 21.28,  *P* < 0.001 (statistically significant).

**Table 3:** API 20E identification of Gram-negative bacilli isolates among positive clinical specimens.

Organism	Total = 65	
	Number	(%)
*Escherichia coli *	30	46.1
*Klebsiella pneumoniae*	17	26.2
*Enterobacter cloacae*	4	6.1
*Pseudomonas aeruginosa*	7	10.7
*Proteus mirabilis*	2	3.07
*Acinetobacter baumanii*	3	4.6
*Citrobacter freundii*	1	1.5
*Proteus vulgaris*	1	1.5

*P* < 0.001 (statistically significant).

**Table 4:** Risk factors associated with isolation of Gram-negative bacilli isolates.

Risk factor	Gram-negative bacilli isolates	Total number of samples	Relative risk (95% CI)^*∗*^	*P* value significance
Age				
<40	26	45	**1.07** (0.78–1.47)	**0.66**
>40	39	63		
Sex				
Male	29	49	**1.09** (0.8–1.47)	**0.59**
Female	36	59		
Trauma				
Yes	48	59	**2.34** (1.57–3.51)	**<0.001**
No	17	49		
Hospitalization length other than ICU				
<7 days	15	51	**2.98** (1.33–4.61)	**<0.001**
>7 days	50	57		
ICU admission				
Yes	28	46	**1.02** (0.45–2.47)	**<0.001**
No	37	62		
Urinary catheter				
Yes	18	32	**0.91** (0.64–1.29)	**0.58**
No	47	76		
Ventilator support				
Yes	10	18	**0.83** (0.51–1.35)	**0.44**
No	20	30		
Central venous catheter				
Yes	4	9	**0.67** (0.31–1.43)	**0.26**
No	26	39		

**∗** = 95% confidence interval.

**Table 5:** Antimicrobial susceptibility patterns by disc diffusion method.

Antibiotic	Antimicrobial susceptibility patterns of all Gram-negative bacilli isolates					
	Susceptible		Intermediate		Resistant	
	Number	(%)	Number	(%)	Number	(%)
Imipenem	31	47.7	1	1.5	33	50.8
Amikacin	40	^*∗*^61.5	1	1.5	24	44.6
Ciprofloxacin	35	53.8	3	4.6	27	41.5
Piperacillin	15	23.1	6	^*∗*^9.2	44	67.7
Cefoperazone/sulbactam	21	32.3	4	6.1	40	61.5
Cefoxitin	12	18.4	0	0.00	53	81.5
Cefotaxime	0	0.00	0	0.00	65	^*∗*^100.00

^*∗*^ *P* < 0.001 (statistically significant).

**Table 6:** The percentage of *bla*_VIM_, *bla*_TEM_,* bla*_SHV_, and *bla*_CTX-M_ genes among Gram-negative bacilli isolates.

Isolates		β-lactamases genes											
Organism	Number	VIM		TEM		SHV		CTX-M-1		CTX-M-9		CTX-M-8/25	
		Number	(%)	Number	(%)	Number	(%)	Number	(%)	Number	(%)	Number	(%)
*Escherichia coli*	15	10	66.6	13	87.0	0	0.0	3	20.0	4	26.6	0	0.00
*Klebsiella pneumonia*	10	8	80.0	9	90.0	5	50	6	60.0	1	10.0	0	0.00
*Enterobacter cloaca*	2	1	50.0	1	50.0	0	0.0	0	0.00	0	0.00	0	0.00
*Pseudomonas aeruginosa *	4	2	50.0	1	25.0	0	0.0	3	75.0	1	25.0	0	0.00
*Proteus mirabilis*	1	0	0.00	0	0.00	0	0.0	0	0.00	0	0.00	0	0.00
*Acinetobacter baumanii *	1	1	100	0	0.00	0	0.0	0	0.00	0	0.00	0	0.00
Total	**33**	**22**	**66.7**	**24**	**72.7 **	**5**	**15.0**	**12**	**36.0 **	**6**	**18.2 **	**0**	**0.00**

## References

[1] Breslow J. Illinois ‘Nightmare Bacteria’ Outbreak Raises Alarms. http://www.pbs.org.

[2] Zowawi H. M., Balkhy H. H., Walsh T. R., Paterson D. L. (2013). *β*-Lactamase production in key gram-negative pathogen isolates from the Arabian Peninsula. *Clinical Microbiology Reviews*.

[3] Kattan J. N., Villegas M. V., Quinn J. P. (2008). New developments in carbapenems. *Clinical Microbiology and Infection*.

[4] Papp-Wallace K. M., Endimiani A., Taracila M. A., Bonomo R. A. (2011). Carbapenems: past, present, and future. *Antimicrobial Agents and Chemotherapy*.

[5] Lee H., Ko K. S., Song J.-H., Peck K. R. (2011). Antimicrobial activity of doripenem and other carbapenems against gram-negative pathogens from Korea. *Microbial Drug Resistance*.

[6] Toleman M. A., Walsh T. R. (2008). Evolution of the ISCR3 group of ISCR elements. *Antimicrobial Agents and Chemotherapy*.

[7] Jemima S. A., Verghese S. (2008). Multiplex PCR for *bla*
_CTX-M_ and *bla*
_SHV_ in the extended spectrum-B-lactamase (ESBL) producing gram-negative isolates. *The Indian Journal of Medical Research*.

[8] Picão R. C., Poirel L., Gales A. C., Nordmann P. (2009). Diversity of *β*-lactamases produced by ceftazidime-resistant *Pseudomonas aeruginosa* isolates causing bloodstream infections in Brazil. *Antimicrobial Agents and Chemotherapy*.

[9] Moland E. S., Black J. A., Ourada J., Reisbig M. D., Hanson N. D., Thomson K. S. (2002). Occurrence of newer *β*-lactamases in *Klebsiella pneumoniae* isolates from 24 U.S. Hospitals. *Antimicrobial Agents and Chemotherapy*.

[10] Dianna L. K., Peter H. G., Patrick R. M., Ellen J. B., James H. J., Miccael A. P., Robert H. Y. (2003). Pseudomonas. *Manual of Clinical Microbiology*.

[11] Clinical and Laboratory Standards Institute (2012). *Performance Standards for Antimicrobial Susceptibility Testing; Twenty-Second Informational Supplement*.

[12] Dallenne C., da Costa A., Decre D., Favier C., Arlet G. (2010). Development of a set of multiplex PCR assays for the detection of genes encoding important *β*-lactamases in *Enterobacteriaceae*. *Journal of Antimicrobial Chemotherapy*.

[13] Altschul S. F., Gish W., Miller W., Myers E. W., Lipman D. J. (1990). Basic local alignment search tool. *Journal of Molecular Biology*.

[14] Pitout J. D. D. (2008). Multiresistant enterobacteriaceae: new threat of an old problem. *Expert Review of Anti-Infective Therapy*.

[15] Manuel J., Martinez R., Nordmann P., Fortineau N., Poirel L. (2010). VIM-19, a metallo-*β*-lactamase with increased carbapenemase activity from *Escherichia coli* and *Klebsiella pneumoniae*. *Antimicrobial Agents and Chemotherapy*.

[16] Rodriguez-Martinez J.-M., Nordmann P., Fortineau N., Poirel L. (2010). VIM-19, a metallo-*β*-lactamase with increased carbapenemase activity from *Escherichia coli* and *Klebsiella pneumoniae*. *Antimicrobial Agents and Chemotherapy*.

[17] Shanthi M., Sekar U. (2010). Extended spectrum beta lactamase producing *Escherichia coli* and *Klebsiella pneumoniae*: risk factors for infection and impact of resistance on outcomes. *Journal of the Association of Physicians of India*.

[18] Benachinmardi K. K., Padmavathy M., Malini J., Naveneeth B. V. (2014). Prevalence of non-fermenting Gram-negative bacilli and their in vitro susceptibility pattern at a tertiary care teaching hospital. *Journal of the Scientific Society*.

[19] Sahu S. K., Dalal A. S., Bansal G. (2011). Detection of extended-spectrum *β*-lactamases in clinical isolates of *E. coli* and *Klebsiella* species from Udaipur Rajasthan. *Biomedical Research*.

[20] Vipin K., Rohit K. M., Avantika C., Pramila G. (2011). Incidence of *β*-lactamase producing gram-negative clinical isolates and their antibiotic susceptibility pattern: a case study in Allahabad. *International Journal of Research in Pure and Applied Microbiology*.

[21] Sankarankutty J., Kaup S. (2014). Microbiological profile and antibiogram of uropathogens from a tertiary care centre in Tumkur, India. *Journal of Microbiology and Biotechnology Research*.

[22] Aboderin O. A., Abdu L.-R., Odetoyin B. W., Lamikanra A. (2009). Antimicrobial resistance in *Escherichia coli* strains from urinary tract infections. *Journal of the National Medical Association*.

[23] Kumar S. H., De A. S., Baveja S. M., Gore M. A. (2012). Prevalence and risk factors of Metallo *β*-lactamase producing *Pseudomonas aeruginosa* and Acinetobacter species in burns and surgical wards in a tertiary care hospital. *Journal of Laboratory Physicians*.

[24] Aktas Z., Satana D., Kayacan C. (2012). Carbapenem resistance in Turkey: repeat report on OXA-48 in *Klebsiella pneumoniae* and first report on IMP-1 beta-lactamase in *Escherichia coli*. *African Journal of Microbiology Research*.

[25] Tuon F. F., Rocha J. L., Toledo P. (2012). Risk factors for KPC-producing *Klebsiella pneumoniae* bacteremia. *Brazilian Journal of Infectious Diseases*.

[26] Prashanth K., Badrinath S. (2004). *In vitro* susceptibility pattern of *Acinetobacter* species to commonly used cephalosporins, quinolones, and aminoglycosides. *Indian Journal of Medical Microbiology*.

[27] Mohamed N. M., Raafat D. (2011). Phenotypic and genotypic detection of metallo-beta-lactamases in imipenem-resistant *Acinetobacter baumannii* isolated from a tertiary hospital in Alexandria, Egypt. *Research Journal of Microbiology*.

[28] Poulou A., Voulgari E., Vrioni G. (2012). Imported *Klebsiella pneumoniae* carbapenemase-producing *K. pneumoniae* clones in a Greek hospital: Impact of infection control measures for restraining their dissemination. *Journal of Clinical Microbiology*.

[29] Al Johani S. M., Akhter J., Balkhy H., El-Saed A., Younan M., Memish Z. (2010). Prevalence of antimicrobial resistance among gram-negative isolates in an adult intensive care unit at a tertiary care center in Saudi Arabia. *Annals of Saudi Medicine*.

[30] Galani I., Rekatsina P. D., Hatzaki D., Plachouras D., Souli M., Giamarellou H. (2008). Evaluation of different laboratory tests for the detection of metallo-*β*-lactamase production in *Enterobacteriaceae*. *Journal of Antimicrobial Chemotherapy*.

[31] Shahcheraghi F., Nikbin V. S., Feizabadi M. M. (2010). Identification and genetic characterization of metallo-beta-lactamase-producing strains of *Pseudomonas aeruginosa* in Tehran, Iran. *New Microbiologica*.

[32] Shahcheraghi F., Abbasalipour M., Feizabadi M. M., Ebrahimipour G. H., Akbari N. (2011). Isolation and genetic characterization of metallo-*β*-lactamase and carbapenamase producing strains of *Acinetobacter baumannii* from patients at tehran hospitals. *Iranian Journal of Microbiology*.

[33] Shah A. A., Hasan F., Ahmed S., Hameed A. (2004). Characteristics, epidemiology and clinical importance of emerging strains of Gram-negative bacilli producing extended-spectrum *β*-lactamases. *Research in Microbiology*.

[34] Eftekhar F., Rastegar M., Golalipoor M., Mansoursamaei N. (2012). Detection of extended spectrum Β-lactamases in urinary isolates of *Klebsiella pneumoniae* in relation to *Bla*
_SHV_, *Bla*
_TEM_ and *Bla*
_CTX-M_ gene carriage. *Iranian Journal of Public Health*.

[35] Ojdana D., Sacha P., Wieczorek P. (2014). The occurrence of *bla*
_CTX-M_, *bla*
_SHV_, and *bla*
_TEM_ genes in extended-spectrum *β*-lactamase-positive strains of *Klebsiella pneumoniae*, *Escherichia coli*, and *Proteus mirabilis* in Poland. *International Journal of Antibiotics*.

[36] Cuzon G., Naas T., Bogaerts P., Glupczynski Y., Nordmann P. (2012). Evaluation of a DNA microarray for the rapid detection of extended-spectrum *β*-lactamases (TEM, SHV and CTX-M), plasmid-mediated cephalosporinases (CMY-2-like, DHA, FOX, ACC-1, ACT/MIR and CMY-1-like/MOX) and carbapenemases (KPC, OXA-48, VIM, IMP and NDM). *Journal of Antimicrobial Chemotherapy*.

[37] Zurfluh K., Hächler H., Nüesch-Inderbinen M., Stephan R. (2013). Characteristics of extended-spectrum *β*-lactamase- and carbapenemase-producing Enterobacteriaceae isolates from rivers and lakes in Switzerland. *Applied and Environmental Microbiology*.

[38] Pokhrel R. H., Thapa B., Kafle R., Shah P. K., Tribuddharat C. (2014). Co-existence of beta-lactamases in clinical isolates of *Escherichia coli* from Kathmandu, Nepal. *BMC Research Notes*.

[39] Alsayed Gaber H., Ahmed M. A., Rania B. M., Mohammed M., Osama M. A. (2014). Mathematical modeling and classification of viruses from herpesvirus family. *International Journal of Computer Applications*.

