# Corrigendum to “Spread of TEM, VIM, SHV, and CTX-M *β*-Lactamases in Imipenem-Resistant Gram-Negative Bacilli Isolated from Egyptian Hospitals”

**DOI:** 10.1155/2017/5959128

**Published:** 2017-09-25

**Authors:** El sayed Hamdy Mohammed, Ahmed Elsadek Fakhr, Hanan Mohammed El sayed, Said abd Elmohsen Al Johery, Wesam Abdel Ghani Hassanein, Ahmed Mansour Alzohairy

**Affiliations:** ^1^Department of Botany & Microbiology, Faculty of Science, Zagazig University, Zagazig 44511, Egypt; ^2^Department of Medical Microbiology & Immunology, Faculty of Medicine, Zagazig University, Zagazig 44511, Egypt; ^3^Department of Genetics, Faculty of Agriculture, Zagazig University, Zagazig 44511, Egypt

In the article titled “Spread of TEM, VIM, SHV, and CTX-M *β*-Lactamases in Imipenem-Resistant Gram-Negative Bacilli Isolated from Egyptian Hospitals” [1], Dr. Ahmed Mansour Alzohairy was missing from the authors' list. The corrected authors' list is shown above. In addition, the Acknowledgments section should be corrected as follows.

## References

[1] Mohammed E. S. H., Elsadek Fakhr A., El Sayed H. M., Al Johery S. A. E., Hassanein W. A. G. (2016). Spread of TEM, VIM, SHV, and CTX-M *β*-lactamases in imipenem-resistant gram-negative bacilli isolated from Egyptian hospitals. *International Journal of Microbiology*.

